# Assessment of redundant randomized clinical trials among patients with ST segment elevation myocardial infarction

**DOI:** 10.1186/s12916-023-02749-2

**Published:** 2023-02-24

**Authors:** Yuanxi Jia, Jun Liang, Wenyao Wang, Xin Wei, Shaoming Xiao, Karen A. Robinson

**Affiliations:** 1 Shenzhen Institute of Advanced Technology, Chinese Academy of Sciences, Shenzhen, China; 2 Blood Station, Tongzhou, Beijing China; 3 Department of Cardiology and Institute of Vascular Medicine, Peking University Third Hospital, Peking University, 49 Huayuan N Rd, Haidian District, Beijing, 100191 China; 4 Department of Cardiology, Virginia Commonwealth University, Richmond, USA; 5 Department of Pediatrics, School of Medicine, Johns Hopkins University, Baltimore, USA; 6 Department of Medicine, School of Medicine, Johns Hopkins University, Baltimore, USA

**Keywords:** Redundant clinical trial, ST segment elevation myocardial infarction, Clinical practice guidelines, China, United States

## Abstract

**Background:**

Redundant clinical trials waste resources and unnecessarily put patients at risk for harm. The objectives of the study were to assess redundant randomized clinical trials (RCTs) conducted in mainland China or the USA among patients with ST segment elevation myocardial infarction (STEMI) and estimate the harm to patients enrolled in redundant RCTs.

**Methods:**

We searched bibliographic databases for eligible RCTs comparing a routine therapy with a placebo or no treatment among patients with STEMI in mainland China or the United States. The routine therapy for STEMI included reperfusion (percutaneous coronary intervention or fibrinolytic therapy), P2Y_12_ receptor inhibitors, statins, and anticoagulants. Redundant RCTs were defined as those initiated or continued recruiting new patients 1 year after the experimental intervention was established as routine therapy in clinical practice guidelines. Cumulative meta-analyses were conducted to confirm the efficacy of these routine therapies.

The primary outcome was the number of extra major adverse cardiac events (MACEs) attributable to the deprivation of routine therapies among patients in the control groups of redundant RCTs—that is, the number of extra MACEs that could have been prevented had these patients received routine therapy.

**Results:**

Nine hundred eighty-three eligible RCTs conducted in mainland China were identified, of which 775 (78.8%) were redundant. None of the five eligible RCTs conducted in the United States were redundant. All redundant RCTs have reiterated the benefits of routine therapies for patients with STEMI, while none were cited by the 2019 clinical practice guideline for the management of STEMI.

The 18,819 patients in the control groups of redundant RCTs experienced 3305 (95% CI: 3169–3441) extra MACEs, including 1091 (1014–1165) deaths, 576 (519–633) recurrent myocardial infarctions, 31 (19–42) revascularizations, 39 (23–54) strokes, 744 (679–810) heart failures, and 823 (754–893) patients with recurrent or exacerbated angina pectoris. Cumulative meta-analyses confirmed the efficacy of the routine therapies among patients in mainland China and supported using practice guidelines to define redundant RCTs.

**Conclusions:**

Redundant RCTs conducted in mainland China have resulted in unnecessary MACEs among patients with STEMI. While the reasons behind redundant RCTs need to be further investigated, these results suggest potential research waste and violation of research ethics.

**Supplementary Information:**

The online version contains supplementary material available at 10.1186/s12916-023-02749-2.

## Background

Randomized clinical trials (RCTs) consume substantial resources and put patients at risk [1, 2]. Therefore, whether an RCT is needed deserves careful consideration. As a prerequisite, new RCTs can be conducted if the current evidence is insufficient or there is uncertainty. Similarly, new RCTs using a placebo or no treatment as control can be initiated if the efficacy of the experimental therapy remains unclear [3]. When the clinical question is settled, new RCTs waste resources and are considered redundant [4, 5]. Moreover, in redundant RCTS, patients in the control group are denied a therapy already known to be effective, raising serious concerns about research ethics [6].

Redundant RCTs have been identified as prevalent in many fields [6–8], although this has been less well studied in developing countries that conduct fewer RCTs. Since 2016, mainland China has been producing more scientific publications than any other country [9], but its fastest-growing output of biomedical research appears to include substantial redundancy [10, 11]. For example, it has been estimated that between 2008 and 2019, more than 2000 redundant RCTs were conducted in mainland China [12]. Those RCTs compared statins, drugs already determined to be beneficial, with placebo or no treatment among patients with coronary artery disease. Patients in the control groups were denied statins and, as a result, may have suffered over 3000 unnecessary major cardiac adverse events (MACEs), including more than 500 deaths, that could have been prevented had they received statins [12].

It remains unclear whether such redundancy is limited to statins and whether other countries conduct redundant RCTs. To partially fill these knowledge gaps, we identified and analyzed redundant RCTs assessing three routine therapies in addition to statins for ST elevation myocardial infarction (STEMI), a type of myocardial infarction characterized by symptoms of myocardial ischemia with persistent electrocardiographic ST elevation and biomarkers of myocardial necrosis [13]. We compared the results between mainland China and the United States (US), the only country that publishes a similar number of scientific publications to mainland China [14].

## Methods

In this meta-research study, we identified eligible RCTs comparing four routine therapies with placebo or no treatment among patients with STEMI, determined which, if any, of these RCTs were redundant, and estimated the extra MACEs caused by the deprivation of routine therapies in redundant RCTs. This study was not subject to institutional review board approval. We followed the Strengthening the Reporting of Observational Studies in Epidemiology (STROBE) reporting guideline for cross-sectional studies [15]. This study was not registered.

### Definition of eligible RCTs

#### Eligible patients

We focused on patients with STEMI. RCTs were excluded if aimed at patients with comorbidities, for example, patients with both STEMI and heart failure. We relied on the diagnosis used in each RCT, i.e., we considered an RCT as recruiting patients with STEMI if it reported recruiting patients with STEMI using its own diagnostic criteria. When the diagnosis was not clearly described in an RCT, we assumed patients to have STEMI if the reduction of elevated ST segment was an outcome in this RCT or if all patients were eligible for fibrinolytic therapy. This assumption was made because only patients with STEMI were eligible for fibrinolytic therapy [16–18].

#### Eligible therapies

We included therapies widely embraced by the clinical community as routine therapies. Specifically, therapies were considered routine if they were strongly (i.e., Recommendation Class I) and consistently recommended by clinical practice guidelines (CPGs) based on sufficient evidence (i.e., Level of Evidence A) in both mainland China and the US.

In April 2021, we screened the CPGs developed by the Chinese Society of Cardiology and the American College of Cardiology/American Heart Association. We identified four routine therapies:

1. Reperfusion, including primary percutaneous coronary intervention (PCI) and fibrinolytic therapy. Fibrinolytic therapy could be performed by tenecteplase, reteplase, alteplase, streptokinase, urokinase, or prourokinase. RCTs evaluating delayed PCI or fibrinolytic therapy were excluded.
2. P2Y_12_ receptor inhibitors, including clopidogrel, ticagrelor, and prasugrel. RCTs evaluating loading P2Y_12_ receptor inhibitors administered before PCI or fibrinolytic therapy were excluded.
3. Statins, including atorvastatin, fluvastatin, lovastatin, pravastatin, rosuvastatin, simvastatin, and pitavastatin. We excluded RCTs evaluating loading statin therapy administered before PCI or fibrinolytic therapy.
4. Anticoagulants, including unfractionated heparin, enoxaparin, fondaparinux, and bivalirudin. We only included RCTs evaluating anticoagulants administered with fibrinolytic therapy.

#### Eligible RCTs

We included RCTs that compared one routine therapy with a placebo or no treatment (standard therapy) for patients with STEMI. Routine therapy might be performed by different types of drugs or operations. For example, reperfusion might be performed by PCI or tenecteplase, while anticoagulation might be performed by unfractionated heparin or enoxaparin. Therefore, we excluded RCTs comparing one type of routine therapy with a placebo/no treatment among patients who all received another type of the same routine therapy. For example, we excluded RCTs comparing PCI with no treatment among patients who all received tenecteplase; we also excluded RCTs comparing enoxaparin with placebo among patients who all received unfractionated heparin.

Randomization could be performed by random number tables, computer programs, flipping a coin, etc. [19]. We also included RCTs that did not specify the randomization method. There was no restriction on the time or setting of the RCTs. We only considered RCTs in which the recruiting centers were exclusively located in mainland China or the US. If the locations of the recruiting centers were not reported, we assumed the recruiting centers of an RCT were exclusively located in mainland China or the US if all the authors were affiliated with institutes that were exclusively located in mainland China or the US.

### Definition of redundant RCTs

Redundant RCTs could be defined by either CPGs or cumulative meta-analyses (CMAs), i.e., RCTs were considered redundant if conducted after the efficacy of routine therapies was confirmed by either CPGs or CMAs. We expected that CPGs would confirm the efficacy of routine therapies later than CMAs [12]. To be more conservative, we used CPGs in the primary analysis to define redundant RCTs. In the sensitivity analyses, we performed CMAs to verify the identification of redundant trials.

#### Entirely redundant RCTs

An RCT was entirely redundant if its recruitment started 1 year after the experimental therapy was established as routine therapy by CPGs for the first time and remained so in the following updates [12]. The 1-year “grace period” allows researchers to learn about the CPGs and terminate their RCTs.

#### Partially redundant RCTs

An RCT was partially redundant if it continued to recruit new patients 1 year after the experimental therapy was established as routine therapy by CPGs for the first time and remained so in the following updates.

#### Cutoff times

As shown in Table 1, the cutoff times defining redundant RCTs in mainland China were identified from two CPGs published in 2007 (for statins) and 2010 (for reperfusion, P2Y_12_ receptor inhibitors, and anticoagulants), while the two CPGs for the US were published in 2004 (for reperfusion) and 2008 (for P2Y_12_ receptor inhibitors, statins, and anticoagulants) [20–23].

**Table 1:** Cutoff times defining redundant RCTs

Therapy	Mainland China		United States	
	Publication of CPGs	Cutoff times	Publication of CPGs	Cutoff times
Reperfusion	August 2010 [20]	August 2011	August 2004 [22]	August 2005
P2Y_12_ receptor inhibitors	August 2010 [20]	August 2011	January 2008 [23]	January 2009
Statins	April 2007 [21]	April 2008	January 2008 [23]	January 2009
Anticoagulants	August 2010 [20]	August 2011	January 2008 [23]	January 2009

The cutoff times for defining redundant RCTs by the therapy and the country. The cutoff times are defined as when a therapy was established as routine therapy in CPGs for the first time and remained so in the following updates

*CPG* clinical practice guidelines, *RCT* randomized clinical trials

### Literature search

We conducted a literature search according to the requirement for systematic reviews and reported search according to the Preferred Reporting Items for Systematic reviews and Meta-Analyses literature search extension (PRISMA-S) [19, 24]. Because a proportion of RCTs conducted in mainland China were published in Chinese journals, we searched two English (PubMed and Embase) plus four Chinese bibliographic databases (the China National Knowledge Infrastructure, Wanfang Data, VIP data, and SinoMed) for eligible RCTs published until June 16, 2021, when the search was conducted [25]. We did not search other sources, such as trial registries, grey literature, references of eligible RCTs, or contact the authors for unpublished RCTs. Only RCTs published as journal articles were considered. The search strategies were developed with a literature search specialist and are listed in Additional file 1: Search Strategy.

Two authors (YJ and LJ) independently screened the title/abstract and full text of the records retrieved from bibliographic databases. Discrepancies were discussed and solved with a third author (WW). We compared similar studies with overlapping authors, facilities recruiting patients, sample size, or outcomes to identify possible duplicates [26].

### Risk of bias assessment

We conducted a risk of bias assessment on MACEs in eligible RCTs according to the Risk of Bias Tool developed by Cochrane [27]. Four types of bias were evaluated: random sequence generation (selection bias), allocation concealment (selection bias), blinding of participants and researchers (performance bias), and blinding of outcome assessment (detection bias). The risk of bias assessment was performed by two authors independently (YJ and JL). Discrepancies were solved with a third author (WW).

### Statistical analysis

#### Descriptive analysis

We reported the characteristics of eligible RCTs, including the number of eligible RCTs, the number of patients recruited, the country where they were conducted, and the result of the risk of bias assessment. We also reported the factors that might be attributed to redundant RCTs, including funding sources, approval from an ethics committee, registration, and conflicts of interest [12]. We classified the conclusions of redundant RCTs as positive (routine therapies were effective and safe) or negative (routine therapies were ineffective or unsafe) based on their conclusions.

As auxiliary analyses, we reported the percentage of redundant RCTs cited in the latest CPGs for the management of STEMI in mainland China and the US [16]. We also reported the percentage of journals publishing redundant RCTs that endorse or follow the recommendations from the International Committee of Medical Journal Editors (ICMJE), as indicated either on their website or in the list maintained by the ICMJE [28].

#### Primary analysis

We expected that the patients in the control group of redundant RCTs would experience more MACEs (referred to as extra MACE) than those in the routine therapy group. The primary outcome was the number of *extra* MACEs experienced by patients who did not receive the routine therapy in redundant RCTs (i.e., patients randomized to the control group). To accommodate the wide range of clinical events reported in individual RCTs, we defined a broad MACE to include all-cause mortality or cardiac-related mortality, recurrent myocardial infarction, stroke, heart failure, revascularization, and recurrent or exacerbated angina pectoris. We relied on the RCTs to define and report these components of MACE.

We estimated the extra MACEs by calculating the risk difference between the intervention and the control group in redundant RCTs—that is, the difference between the observed incidence of MACEs in the control group and the expected incidence of MACEs if patients in the control group had been treated as in the routine therapy group. In other words, the extra MACEs could have been prevented if patients in the control group had been given the routine therapy.

We only considered the extra MACE that occurred in the redundant phase of eligible RCTs. While all extra MACEs in entirely redundant RCTs were considered, in partially redundant RCTs, only those that occurred after the cutoff times were considered, assuming a smooth rate during the study period. We used bootstrapping to construct the 95% confidence intervals of extra MACEs (Additional file 2: Additional Method). The primary analysis was performed in SAS 9.4.

#### Sensitivity analyses

We performed CMAs to verify the assumption that CMAs confirmed the efficacy of routine therapies earlier than CPGs. A year was considered the cutoff time if the 95% confidence interval of the cumulative risk difference did not cross zero for the first time this year and remained so in the following years. We planned to conduct CMAs separately for mainland China and the US. However, because only five eligible RCTs were from the US, the CMAs were only performed among eligible RCTs from mainland China. CMAs were conducted using Stata 16.

## Results

### Characteristics of redundant RCTs

We identified 983 eligible RCTs conducted in mainland China (Additional file 3: Figure A1), of which 775 (78.8%) were redundant, including 631 (64.2%) entirely redundant RCTs and 144 (14.6%) partially redundant RCTs (Table 2). A total of 45,024 patients were recruited in eligible RCTs, 35,930 (79.8%) of which were recruited in redundant RCTs. Most redundant RCTs assessed reperfusion (366 RCTs, 47.2% of redundant RCTs), followed by P2Y_12_ receptor inhibitors (247, 31.9%), and statins (126, 16.3%). Similarly, redundant RCTs assessing reperfusion recruited the most patients (16,462 patients, 45.8% of all patients in redundant RCTs), followed by P2Y_12_ receptor inhibitors (11,520, 32.1%), and statins (6211, 17.3%). Only 122 (12.4%) eligible RCTs conducted in mainland China reported approval from an ethics committee, 23 (2.3%) reported funding sources, and 541 (55.0%) reported MACE as an outcome.

**Table 2:** Characteristics of eligible RCTs

Category	Mainland China					United States
	Non-redundant	Redundant			Total	Non-redundant
		Partially redundant	Entirely redundant	All redundant		
**No. of RCTs (%)**						
Total	208 (21.2)	144 (14.6)	631 (64.2)	775 (78.8)	983 (100.0)	5 (100.0)
Reperfusion	147 (28.7)	72 (14.0)	294 (57.3)	366 (71.3)	513 (100.0)	3 (100.0)
P2Y_12_ receptor inhibitors	33 (11.8)	41 (14.6)	206 (73.6)	247 (88.2)	280 (100.0)	0 (0.0)
Statins	13 (26.5)	7 (14.3)	29 (59.2)	36 (73.5)	49 (100.0)	2 (100.0)
Anticoagulants	15 (10.6)	24 (17.0)	102 (72.3)	126 (89.4)	141 (100.0)	0 (0.0)
**No. of patients recruited (%)**						
Total	9094 (20.2)	7389 (16.4)	28,541 (63.4)	35,930 (79.8)	45,024 (100.0)	284 (100.0)
Reperfusion	6082 (27.0)	3635 (16.1)	12,827 (56.9)	16,462 (73.0)	22,544 (100.0)	120 (100.0)
P2Y_12_ receptor inhibitors	1891 (14.1)	2031 (15.1)	9489 (70.8)	11,520 (85.9)	13,411 (100.0)	0 (0.0)
Statins	583 (25.1)	385 (16.6)	1352 (58.3)	1737 (74.9)	2320 (100.0)	164 (100.0)
Anticoagulants	538 (8.0)	1338 (19.8)	4873 (72.2)	6211 (92.0)	6749 (100.0)	0 (0.0)
**Ethics committee approval**						
Reported	1 (0.5)	2 (1.4)	119 (18.9)	121 (15.6)	122 (12.4)	3 (60.0)
Not reported	207 (99.5)	142 (98.6)	509 (80.7)	651 (84.0)	858 (87.3)	2 (40.0)
**Funding sources**						
Government	3 (1.4)	0 (0.0)	14 (2.2)	14 (1.8)	17 (1.7)	1 (20.0)
Industry	0 (0.0)	0 (0.0)	0 (0.0)	0 (0.0)	0 (0.0)	2 (40.0)
Other	1 (0.5)	0 (0.0)	5 (0.8)	5 (0.6)	6 (0.6)	0 (0.0)
Not reported	204 (98.1)	144 (100.0)	612 (97.0)	756 (97.5)	960 (97.7)	2 (40.0)
**MACE as an outcome**						
Reported	151 (72.6)	93 (64.6)	297 (47.1)	390 (50.3)	541 (55.0)	4 (80.0)
Not reported	57 (27.4)	51 (35.4)	334 (52.9)	385 (49.7)	442 (45.0)	1 (20.0)

The characteristics of eligible RCTs presented by the country (mainland China vs. the US) and the redundancy (non-redundant vs. partially redundant vs. entirely redundant)

*RCT* randomized clinical trials, *MACE* major adverse cardiac events

Meanwhile, we identified five eligible RCTs that were conducted in the US; none was redundant. The five RCTs recruited 284 patients in control groups before relevant cutoff times for defining redundant trials and recruited none after. Three eligible RCTs conducted in the US reported an approval from an ethics committee, three reported funding sources, and four reported MACE as an outcome.

None of the redundant RCTs reported conflicts of interest or were registered in a trial registry. Except for two published in English-language journals, 775 redundant RCTs were published in 138 Chinese-language journals. None of the 140 journals stated following the recommendations from the ICMJE, either on their website or in the list maintained by the ICMJE.

**Fig. 1:**
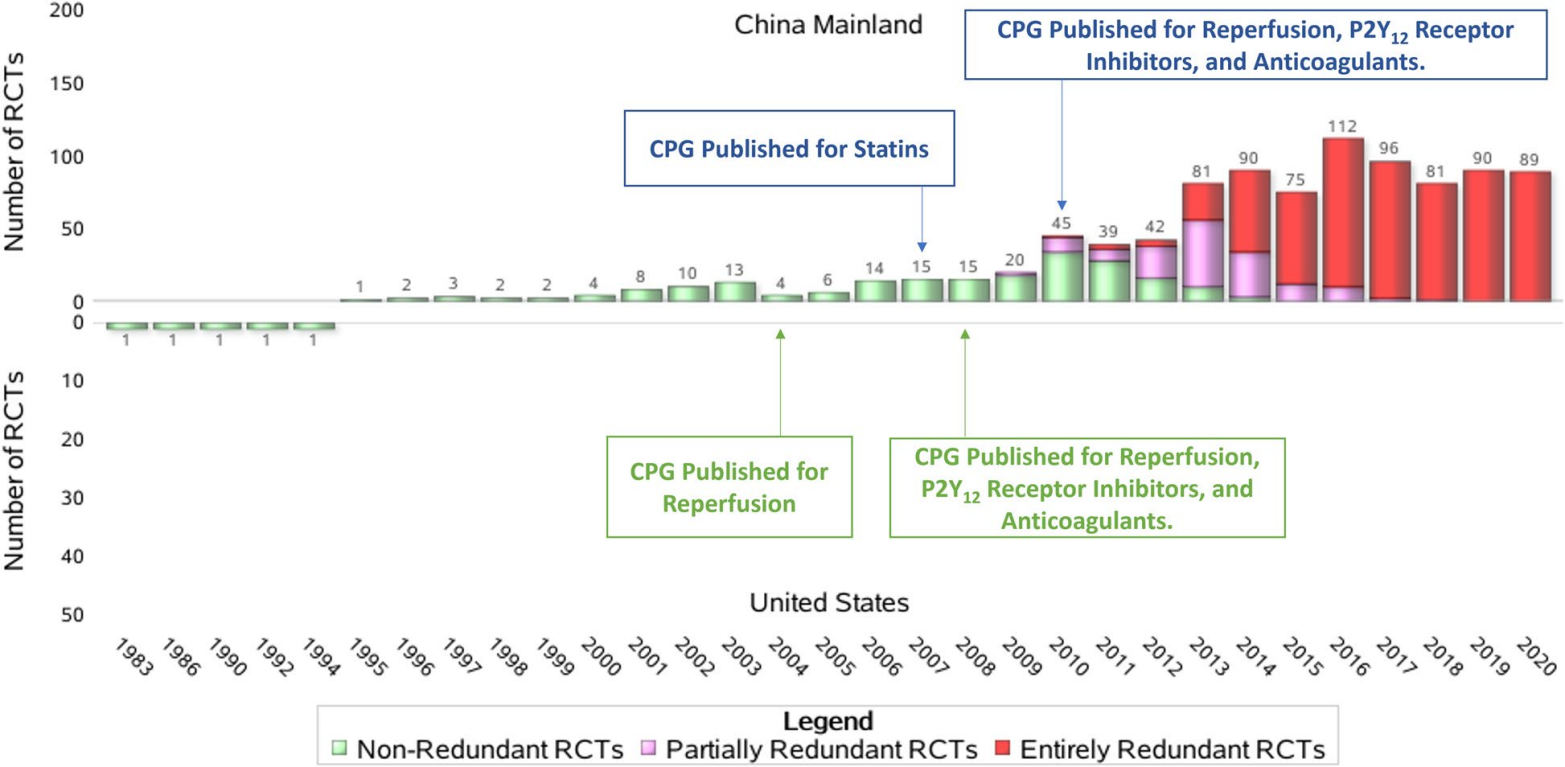
Distribution of included RCTs by year of publishing. China Mainland vs. the US. The distribution of eligible RCTs by year of publishing for mainland China and the US. CPG, clinical practice guideline; RCT, randomized clinical trial

**Fig. 2:**
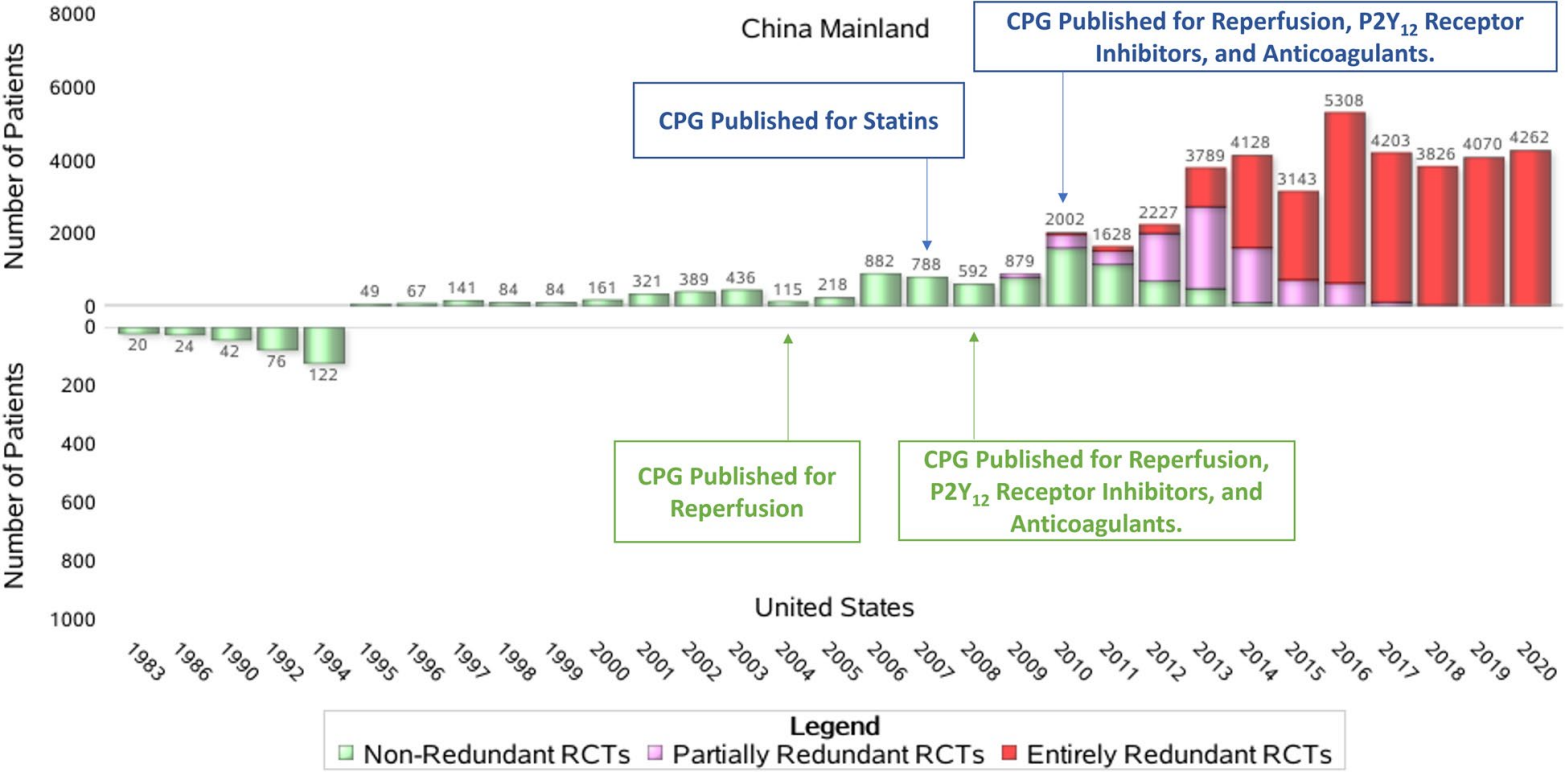
Distribution of patients recruited in the control group by year of publishing. China Mainland vs. the US. The distribution of the patients recruited in the control group of eligible RCTs by year of publishing for mainland China and the US. CPG, clinical practice guideline; RCT, randomized clinical trial

Figure 1 shows the distribution of redundant and non-redundant RCTs over time in the US and mainland China. All five RCTs from the US were published before 1995—before their first corresponding RCT in mainland China was published. Starting in 2009, the number of redundant RCTs from mainland China increased and reached a peak in 2016, subsequently decreasing in number and leveling off through 2020—a pattern paralleled by the number of control patients (Fig. 2).

All 775 redundant RCTs concluded that the routine therapies were superior to the control, based on either clinical outcomes or surrogate outcomes. None was cited in the subsequent Chinese CPG for the management of STEMI in 2019.

The result of the risk of bias assessment is shown in Table 3. The risk of bias from random sequence generation was rated as low in 90 (16.6%) redundant RCTs compared with 4 (2.6%) non-redundant RCTs conducted in mainland China. However, the risk of bias from allocation concealment was rated as unclear, while the risk of bias from blinding of participants and researchers and blinding of outcome assessment was rated as high in most eligible RCTs conducted in mainland China, regardless of redundancy.

**Table 3:** Risk of bias assessment on MACE in eligible RCTs

Domain	Mainland China					United States
	Non-redundant RCTs	Redundant RCTs			Total	Non-redundant RCTs
		Partially redundant RCTs	Entirely redundant RCTs	Total		
No. of RCTs reporting MACE	151	93	297	390	541	4
**Random sequence generation**						
Low Risk	4 (2.6)	7 (7.5)	79 (26.6)	86 (22.1)	90 (16.6)	1 (25.0)
High risk	0 (0.0)	0 (0.0)	0 (0.0)	0 (0.0)	0 (0.0)	0 (0.0)
Unclear risk	147 (97.4)	86 (92.5)	218 (73.4)	304 (77.9)	451 (83.4)	3 (75.0)
**Allocation concealment**						
Low risk	0 (0.0)	0 (0.0)	0 (0.0)	0 (0.0)	0 (0.0)	2 (50.0)
High risk	0 (0.0)	1 (1.1)	14 (4.7)	15 (3.8)	15 (2.8)	0 (0.0)
Unclear risk	151 (100.0)	92 (98.9)	283 (95.3)	375 (96.2)	526 (97.2)	2 (50.0)
**Blinding of participants and researchers**						
Low risk	0 (0.0)	0 (0.0)	0 (0.0)	0 (0.0)	0 (0.0)	0 (0.0)
High risk	147 (97.4)	92 (98.9)	292 (98.3)	384 (98.5)	531 (98.2)	3 (75.0)
Unclear risk	4 (2.6)	1 (1.1)	5 (1.7)	6 (1.5)	10 (1.8)	1 (0.0)
**Blinding of outcome assessment**						
Low risk	0 (0.0)	0 (0.0)	0 (0.0)	0 (0.0)	0 (0.0)	1 (25.0)
High risk	148 (98.0)	92 (98.9)	292 (98.3)	384 (98.5)	532 (98.3)	2 (50.0)
Unclear risk	3 (2.0)	1 (1.1)	5 (1.7)	6 (1.5)	9 (1.7)	1 (25.0)

The result of the risk of bias assessment on MACE in eligible RCTs

*RCT* randomized clinical trials, *MACE* major adverse cardiac events

### Primary outcome

The MACEs were reported as an outcome in 390 of 775 (50.3%) redundant RCTs. The 18,819 patients in the control groups of redundant RCTs experienced 3305 (95%CI: 3169 to 3441) extra MACEs, including 1091 (1014 to 1165) deaths, 576 (519 to 633) recurrent myocardial infarctions, 31 (19 to 42) revascularizations, 39 (23 to 54) strokes, 744 (679 to 810) heart failures, 823 (754 to 893) recurrent or exacerbated angina pectoris, and 4 (2 to 7) unspecified MACEs (Table 4). The redundant RCTs assessing reperfusion reported the most extra MACEs (1535, 46.4%), followed by P2Y_12_ receptor inhibitors (914, 27.7%), and statins (631, 19.1%). Similarly, the redundant RCTs assessing reperfusion reported the most extra deaths (617, 56.6%), followed by P2Y_12_ receptor inhibitors (253, 23.2%), and statins (159, 14.6%).

**Table 4:** Number of extra MACEs in the control groups

Data items	Routine Therapy				Total
	Reperfusion	P2Y_**12**_ receptor inhibitors	Statins	Anticoagulants	
No. of redundant RCTs (%)	210 (53.8)	109 (27.9)	59 (15.1)	12 (3.1)	390 (100.0)
No. of patients in the control group (%)	9714 (51.6)	5379 (28.6)	3,039 (16.1)	687 (3.7)	18819 (100.0)
Extra MACEs (95%CI)					
Death	617 (562, 672)	253 (215, 290)	159 (129, 189)	62 (45, 80)	1091 (1014, 1165)
Myocardial infarction	154 (127, 182)	251 (213, 287)	161 (129, 193)	10 (2, 19)	576 (519, 633)
Revascularization	0 (0, 0)	20 (10, 29)	12 (5, 18)	0 (0, 0)	31 (19, 42)
Stroke	0 (0, 0 )	10 (2, 18)	29 (17, 41)	0 (0, 0)	39 (23, 54)
Heart failure	422 (372, 472)	85 (61, 110)	123 (96, 148)	116 (94, 139)	744 (679, 810)
Angina pectoris	343 (299, 387)	294 (253, 335)	149 (119, 180)	38 (23, 54)	823 (754, 893)
Unspecified MACEs	0 (0, 0)	4 (2, 7)	0 (0, 0)	0 (0, 0)	4 (2, 7)
Total	1535 (1,442, 1,625)	914 (842, 985)	631 (570, 692)	226 (192, 260)	3305 (3169, 3441)

The number of patients recruited in redundant RCTs and the extra MACEs experienced by the patients recruited in the control groups of redundant RCTs. The extra MACEs are attributable to the deprivation of routine therapies—that is, the number of extra MACEs that could have been prevented had those patients received routine therapy

*MACE* major adverse cardiac event, *RCT* randomized clinical trial

### Sensitivity analysis

We performed CMAs for eligible RCTs conducted in mainland China. The CMAs showed that reperfusion, P2Y_12_ receptor inhibitors, statins, and anticoagulants could significantly reduce the incidence of death and recurrent myocardial infarction among Chinese patients with STEMI by 1996 (Additional file 4: Figure A2), 2007 (Additional file 5: Figure A3), and 2006 (Additional file 6: Figure A4), respectively, and remained so in the following years. As expected, the times when CMAs confirmed the efficacy of reperfusion, P2Y_12_ receptor inhibitors, and statins were before the corresponding CPGs were published. The only exception was anticoagulants, for which the efficacy was confirmed by both CPGs and CMAs in 2010 in mainland China (Additional file 7: Figure A5) and remained so in the following years.

## Discussion

### Main findings

This study revealed the massive scale of redundant RCTs assessing four routine therapies among patients with STEMI conducted in China. These redundant RCTs wasted resources and only reiterated prior findings and had a limited impact on CPGs. Moreover, the conduct of these redundant RCTs led to over 3000 unnecessary MACEs that could have been prevented had those patients not been recruited.

This study illustrates the massive redundancy of RCTs in mainland China. Moreover, such redundancy was not found in the US. The temporal distribution of redundant RCTs identified in the current study was similar to our previous study on statins [12]. Although it remains unclear why the number of redundant RCTs peaked in 2016, the overall temporal trend implies that more redundant RCTs are likely to be initiated soon unless drastic measures are taken immediately by stakeholders.

### Strength of the study

We applied a series of conservative methods to reduce controversies and improve the credibility of the results. First, we used CPGs to identify the cutoff times to define redundant RCTs rather than CMAs. Although both CPGs and CMAs could be used to identify the cutoff times [12, 29, 30], those identified by CMAs were earlier and more aggressive than those by CPGs [12]. This study reiterated that more redundant RCTs would have been identified if CMAs had been used to identify the cutoff times. Second, we added a 1-year grace period for researchers to learn the CPGs and terminate their RCTs, leading to fewer redundant RCTs. Third, we assessed the redundancy of eligible RCTs for mainland China and the US separately. When developing CPGs, the expert committee usually considers all available evidence, regardless of the country where they were conducted. Similarly, the RCTs in the US should be considered when assessing the redundancy of the RCTs conducted in mainland China. However, the efficacy or safety of some therapies may differ across countries due to genetic or economic factors [31–33]. Even though some therapies have already been confirmed as beneficial and safe in the US, bridging RCTs reaffirming their benefits and safety in the Chinese population can still be justified. Therefore, assessing the redundancy of eligible RCTs for mainland China and the US separately would rule out the potential justification of country-level diversity and result in more conservative but less controversial results.

We did not restrict the demographic characteristics of patients recruited in eligible RCTs. Therefore, redundant RCTs may be diverse in patients' demographic characteristics. However, this would not affect the validity of the results and conclusions of our study for two reasons. First, CPGs recommended routine therapies for all patients with STEMI without contradictions, regardless of demographic characteristics. Second, there were no signs of significant heterogeneity in the effect of routine therapies in a specific population. As shown in this study, all redundant RCTs have reiterated the efficacy and safety of routine therapies.

We relied on the RCTs to define and report the components of MACEs, of which the diagnosis criteria may vary across RCTs, leading to misclassifying some MACEs. However, this non-differential misclassification would underestimate the risk difference between the routine therapy group and the control group in RCTs and, thus, underestimate the extra MACEs. As a result, the validity of the results or conclusions of this study would not be affected.

### Reasons behind the redundancy

Scientific publications are used to evaluate clinician performance for promotion in mainland China. The need for promotion, in some settings, has become a major incentive to conduct clinical research [34, 35]. Under this pressure, clinicians with inadequate training in clinical trials may conduct RCTs for the sole purpose of publication [34, 36]. Pharmaceutical companies could also be involved, although that is difficult to determine as none of the redundant RCTs disclosed conflicts of interest and few reported funding sources. A lack of training of junior clinicians on conducting ethical and legitimate clinical research may also play a key role [34, 36]. For example, very few redundant RCTs identified in this study cited systematic reviews or CPGs. Had the authors of redundant RCTs examined prior systematic reviews or CPGs before initiating the RCTs, they would have known the lack of justification for performing the RCTs.

Recently, the Chinese government has been trying to change the focus from quantity to quality of publications. This endeavor can potentially increase the scientific value of clinical publications, reduce waste, and save lives [37]. Although there has been dispute over whether the conduct of clinical research should be required for all clinicians in mainland China [38], offering those not capable of or not interested in conducting clinical research the option to focus on clinical practice may help reduce redundant trials.

To be conducted and published, a redundant RCT must pass supervision from several stakeholders. Therefore, the scale of redundancy we found implies oversight failures throughout the research system. First, 15.6% of redundant RCTs reported approval from an ethics committee, implying that those ethics committees failed in their responsibility to protect patients. We could not determine why approval from an ethics committee was not reported in the rest of the redundant RCTs. Second, 2.4% of redundant RCTs reported funding sources, implying that those funders failed to scrutinize the scientific merits of the redundant RCTs. Sometimes, the funders may find it challenging to assess each research proposal’s scientific merit thoroughly. However, they could request applicants provide a summary of prior evidence, e.g., a systematic review, as a prerequisite for approval, which could help reduce research waste [39]. Third, trial registries can display the completed and ongoing clinical trials to the research community and thus reduce waste [40, 41]. Unfortunately, none of the redundant RCTs were registered, leaving the research community unaware of those RCTs until their publication. This finding was consistent with our prior study [12]. Fourth, none of the 140 journals publishing the redundant RCTs stated they followed the ICMJE. Some of these journals might pursue profit rather than scientific merits, including so-called predatory journals, which usually fail to perform peer-review [42].

### Limitations

There are several limitations of this study. First, we may have underestimated the number of redundant RCTs and the unnecessary MACEs because we only relied on journal articles. More than half of redundant RCTs did not report MACEs, while some redundant RCTs may never be published or reported only in grey literature. In addition, we could not determine the relevancy of many journal articles because of missing key information.

Second, we may have overestimated the number of redundant RCTs and the unnecessary MACEs because we took the journal articles at their face value. Evidence suggests that the methods and results reported in journal articles may deviate from the actual conduct [43–45]. For example, a cohort study might be reported as an RCT. In addition, the publications of some redundant RCTs shared similar wording and structure, which may indicate that these trials were partially or entirely fabricated by either individual researchers or essay mills, the companies producing faked publications for profit. However, we did not assess this suspicion further. Although the pre-clinical research field has already been polluted by partially or entirely faked publications [46], it remains unclear whether such misconduct has seeped into clinical research in mainland China.

When cohort studies are misreported as RCTs, the unnecessary MACEs could still be attributed to the deprivation of routine therapies because the patients in the control groups were reportedly comparable to those in the routine therapy group and eligible for routine therapies without contraindications. If the journal articles were partially or entirely fabricated, the number of redundant RCTs and unnecessary MACEs could have been overestimated.

Third, we only searched PubMed and Embase for eligible RCTs published in English journals. We did not search the Cochrane Central Register of Controlled Trials (CENTRAL) because CENTRAL did not provide the function to search the records by authors’ affiliations, while we used keywords such as “China” or “United States” to restrict the authors’ affiliations when searching PubMed and Embase. A recent study showed that the combination of PubMed and Embase could cover almost 90% of studies included in the Cochrane reviews [47]. Therefore, we were confident that most eligible RCTs were identified; the few we might have missed would not significantly impact the results and conclusions of this study.

Fourth, we relied on the diagnosis of STEMI in the eligible RCTs. The diagnostic criteria adopted in redundant RCTs were generally similar to those defined in CPGs, while the details may vary. The deviation from CPGs may lead to the inclusion of patients in redundant RCTs who should not be considered STEMI. However, this would not affect the validity of the results and conclusions from our study. The similarity of diagnosis criteria between eligible RCTs and CPGs implies that a proportion of patients in redundant RCTs still had “true” STEMI. Therefore, the authors should not conduct placebo or standard care controlled RCTs and deprive those patients of routine therapies. If researchers planned to assess the benefits of routine therapies for the patients who had STEMI by their own criteria but not by the CPGs criteria, those patients who had STEMI by the CPGs criteria should be excluded from the RCTs. Unfortunately, none of the redundant RCTs stated that.

Fifth, the quality of eligible RCTs conducted in mainland China was generally low, including those included in the CMAs. Therefore, the result of CMAs based on these high- or unclear-risk RCTs may not be valid. However, this would not impact the validity of the definition of redundant RCTs. Suppose redundant RCTs, which were published later, were initiated to address the low quality of non-redundant RCTs. In that case, the quality of redundant RCTs should be significantly better than non-redundant RCTs. Unfortunately, the risk of bias assessment indicated that the quality of redundant RCTs was also low. Therefore, the low quality of non-redundant RCTs could not justify the initiation of redundant RCTs.

### Future directions

Although our study has shown the large scale of redundant RCTs in mainland China, the reasons behind this phenomenon remain unclear. We recommend in-depth surveys or qualitative studies of the stakeholders, including patients participating in the redundant RCTs, the authors of redundant RCTs, the ethics committees, the funders, and the journal editors. If cohort studies were reported as RCTs, we need to understand why the patients in the control groups did not receive the routine therapies, e.g., patients’ refusal due to personal belief or financial difficulty, clinician ignorance or negligence, or a shortage of medication supplies. There is a need to develop methods to identify errors in journal articles, including intentional errors and even if articles were partially or entirely fabricated [48]. Finally, the reporting quality of clinical trials in mainland China has to be improved to facilitate future research and to facilitate the use of the trial results in CPGs.

The Chinese government has been making substantial efforts to reduce research redundancy. For example, the Drug Clinical Trial Registry Platform was established in 2012, where all commercial trials supervised by the National Medical Products Administration are required to be registered [49]. The National Health Commission has been updating the ethical standards for investigator-initiated clinical trials [50]. The Chinese government could ensure that the current laws and regulations are followed while expanding their efforts to involve additional stakeholders. For example, trial registration should be mandated for all clinical trials, as being required by the updated Declaration of Helsinki [51]; the public funders should focus more on the scientific merits of proposals; the training on clinicians for conducting ethical and high-quality research should be reinforced; and the evaluation on the performance of clinicians should be balanced between research and clinical practice.

Our study only assessed redundant RCTs in cardiology in mainland China and the US. It is unclear whether the results apply to other clinical fields or countries, including the scope of redundancy, the harm to patients, or the reasons for redundancy. Future studies are needed to explore potential redundant RCTs in other clinical fields or countries on a case-by-case basis. Although CPGs were used to define redundant RCTs in this study, we did not imply that all recommendations in CPGs, regardless of the evidence base, should be followed without further examination. Researchers are encouraged to assess the validity of recommendations in CPGs without adequate evidence.

## Conclusions

A total of 775 redundant RCTs recruiting patients with STEMI have been conducted in mainland China; no redundant trials were identified from the US. These redundant RCTs may have led to over 3000 extra MACEs, including 1091 deaths, that could have been prevented had these RCTs were not conducted. While the reasons behind redundant RCTs need to be further investigated, these results suggest potential research waste and a violation of research ethics.

## Supplementary Information

**Additional file 1.** Search Strategy.

**Additional file 2: Additional Method.** The steps to calculate the confidence interval of extra MACEs are presented.

**Additional file 3: Figure A1.** The steps to select Eligible RCTs from bibliographic databases are presented.

**Additional file 4: Figure A2.** Cumulative Meta-Analysis for Reperfusion. This figure shows the result of a cumulative meta-analysis for RCTs assessing reperfusion conducted in mainland China. Only the first 50 RCTs were analyzed due to the limit of Stata.

**Additional file 5: Figure A3.** Cumulative Meta-Analysis for P2Y12 Receptor Inhibitors. This figure shows the result of a cumulative meta-analysis for RCTs assessing P2Y12 receptor inhibitors conducted in mainland China. Only the first 50 RCTs were analyzed due to the limit of Stata.

**Additional file 6: Figure A4.** Cumulative Meta-Analysis for Statins. This figure shows the result of a cumulative meta-analysis for RCTs assessing statins conducted in mainland China. Only the first 50 RCTs were analyzed due to the limit of Stata.

**Additional file 7: Figure A5.** Cumulative Meta-Analysis for Anticoagulants. This figure shows the result of a cumulative meta-analysis for RCTs assessing anticoagulants conducted in mainland China.

## Data Availability

The data are available by emailing Yuanxi Jia at yx.jia@siat.ac.cn.

## Abbreviations

CENTRAL: Cochrane Central Register of Controlled Trials
CMA: Cumulative meta-analysis
CPG: Clinical practice guideline
ICMJE: International Committee of Medical Journal Editors
MACE: Major adverse cardiac events
PCI: Percutaneous coronary intervention
PRISMA-S: Preferred Reporting Items for Systematic reviews and Meta-Analyses literature search extension
RCT: Randomized clinical trial
STEMI: ST segment elevation myocardial infarction
STROBE: Strengthening the Reporting of Observational Studies in Epidemiology
US: United States
