# Supplementary material for: Assessment of redundant randomized clinical trials among patients with ST segment elevation myocardial infarction
Source: BMC Med. 2023 Feb 24;21:69. doi: 10.1186/s12916-023-02749-2 (PMC9960404; doi:10.1186/s12916-023-02749-2)
Supplement: Supplementary file 1 — Additional file 1. Search Strategy. [file 12916_2023_2749_MOESM1_ESM.docx]

Additional File 1 Search Strategy

**PubMed**

(Myocardial infarct*[ti] OR Heart Attack*[ti])

AND

(randomized controlled trial[pt] OR controlled clinical trial[pt] OR randomized[tiab] OR placebo[tiab] OR clinical trials as topic[mesh:noexp] OR randomly[tiab] OR trial[ti] NOT (animals[mh] NOT humans [mh]))

AND

English[la]

AND

(China[AD] OR PRC[AD] OR P.R.C[AD] OR Beijing[AD] OR Tianjin[AD] OR Shanghai[AD] OR Chongqing[AD] OR Hebei[AD] OR Shanxi[AD] OR Liaoning[AD] OR Jilin[AD] OR Heilongjiang[AD] OR Jiangsu[AD] OR Zhejiang[AD] OR Anhui[AD] OR Fujian[AD] OR Jiangxi[AD] OR Shandong[AD] OR Henan[AD] OR Hubei[AD] OR Hunan[AD] OR Guangdong[AD] OR Hainan[AD] OR Sichuan[AD] OR Guizhou[AD] OR Yunnan[AD] OR Shaanxi[AD] OR Gansu[AD] OR Qinghai[AD] OR Inner Mongolia[AD] OR Neimenggu[AD] OR Guangxi[AD] OR Tibet[AD] OR Xizang[AD] OR Ningxia[AD] OR Xinjiang[AD] OR Peking[AD] OR Bei Jing[AD] OR Tian Jin[AD] OR Shang Hai[AD] OR Chong Qing[AD] OR He Bei[AD] OR Shan Xi[AD] OR Liao Ning[AD] OR Ji Lin[AD] OR Hei Long Jiang[AD] OR Jiang Su[AD] OR Zhe Jiang[AD] OR An Hui[AD] OR Fu Jian[AD] OR Jiang Xi[AD] OR Shan Dong[AD] OR He Nan[AD] OR Hu Bei[AD] OR Hu Nan[AD] OR Guang Dong[AD] OR Hai Nan[AD] OR Si Chuan[AD] OR Gui Zhou[AD] OR Yun Nan[AD] OR Shaan Xi[AD] OR Gan Su[AD] OR Qing Hai[AD] OR Nei Meng Gu[AD] OR Guang Xi[AD] OR Tibet[AD] OR Xi Zang[AD] OR Ning Xia[AD] OR Xin Jiang[AD]

OR

USA[AD] OR US[AD] OR United States[AD] OR U.S.[AD] OR U.S.A.[AD] OR Alabama[AD] OR AL[AD] OR Ala.[AD] OR Alaska[AD] OR AK[AD] OR Alaska[AD] OR Arizona[AD] OR AZ[AD] OR Ariz.[AD] OR Arkansas[AD] OR AR[AD] OR Ark.[AD] OR California[AD] OR CA[AD] OR Calif.[AD] OR Colorado[AD] OR CO[AD] OR Color.[AD] OR Connecticut[AD] OR CT[AD] OR Conn.[AD] OR Delaware[AD] OR DE[AD] OR Del.[AD] OR Florida[AD] OR FL[AD] OR Fla.[AD] OR Georgia[AD] OR GA[AD] OR Ga.[AD] OR Hawaii[AD] OR HI[AD] OR Hawaii[AD] OR Idaho[AD] OR ID[AD] OR Idaho[AD] OR Illinois[AD] OR IL[AD] OR Ill.[AD] OR Indiana[AD] OR IN[AD] OR Ind.[AD] OR Iowa[AD] OR IA[AD] OR Iowa[AD] OR Kansas[AD] OR KS[AD] OR Kan.[AD] OR Kentucky[AD] OR KY[AD] OR Ky.[AD] OR Louisiana[AD] OR LA[AD] OR La.[AD] OR Maine[AD] OR ME[AD] OR Maine[AD] OR Maryland[AD] OR MD[AD] OR Md.[AD] OR Massachusetts[AD] OR MA[AD] OR Mass.[AD] OR Michigan[AD] OR MI[AD] OR Mich.[AD] OR Minnesota[AD] OR MN[AD] OR Minn.[AD] OR Mississippi[AD] OR MS[AD] OR Miss.[AD] OR Missouri[AD] OR MO[AD] OR Mo.[AD] OR Montana[AD] OR MT[AD] OR Mont.[AD] OR Nebraska[AD] OR NE[AD] OR Neb.[AD] OR Nevada[AD] OR NV[AD] OR Nev.[AD] OR New Hampshire[AD] OR NH[AD] OR N.H.[AD] OR New Jersey[AD] OR NJ[AD] OR N.J.[AD] OR New Mexico[AD] OR NM[AD] OR N.M.[AD] OR New York[AD] OR NY[AD] OR N.Y.[AD] OR North Carolina[AD] OR NC[AD] OR N.C.[AD] OR North Dakota[AD] OR ND[AD] OR N.D.[AD] OR Ohio[AD] OR OH[AD] OR Ohio[AD] OR Oklahoma[AD] OR OK[AD] OR Okla.[AD] OR Oregon[AD] OR OR[AD] OR Ore.[AD] OR Pennsylvania[AD] OR PA[AD] OR Pa.[AD] OR Rhode Island[AD] OR RI[AD] OR R.I.[AD] OR South Carolina[AD] OR SC[AD] OR S.C.[AD] OR South Dakota[AD] OR SD[AD] OR S.Dak.[AD] OR Tennessee[AD] OR TN[AD] OR Tenn.[AD] OR Texas[AD] OR TX[AD] OR Tex.[AD] OR Utah[AD] OR UT[AD] OR Utah[AD] OR Vermont[AD] OR VT[AD] OR V.T.[AD] OR Virginia[AD] OR VA[AD] OR Va.[AD] OR Washington[AD] OR WA[AD] OR Wash.[AD] OR West Virginia[AD] OR WV[AD] OR W.Va.[AD] OR Wisconsin[AD] OR WI[AD] OR Wis.[AD] OR Wyoming[AD] OR WY[AD] OR Wyo.[AD])

**Embase**

(‘Myocardial infarct*’ OR ‘Heart Attack*’):ti

AND

(random*:ab,ti OR placebo*:de,ab,ti OR (double NEXT/1 blind*):ab,ti)

AND

English:la

AND

(‘China’ OR ‘PRC’ OR ‘P.R.C’ OR ‘Beijing’ OR ‘Tianjin’ OR ‘Shanghai’ OR ‘Chongqing’ OR ‘Hebei’ OR ‘Shanxi’ OR ‘Liaoning’ OR ‘Jilin’ OR ‘Heilongjiang’ OR ‘Jiangsu’ OR ‘Zhejiang’ OR ‘Anhui’ OR ‘Fujian’ OR ‘Jiangxi’ OR ‘Shandong’ OR ‘Henan’ OR ‘Hubei’ OR ‘Hunan’ OR ‘Guangdong’ OR ‘Hainan’ OR ‘Sichuan’ OR ‘Guizhou’ OR ‘Yunnan’ OR ‘Shaanxi’ OR ‘Gansu’ OR ‘Qinghai’ OR ‘Inner Mongolia’ OR ‘Neimenggu’ OR ‘Guangxi’ OR ‘Tibet’ OR ‘Xizang’ OR ‘Ningxia’ OR ‘Xinjiang’ OR ‘Peking’ OR ‘Bei Jing’ OR ‘Tian Jin’ OR ‘Shang Hai’ OR ‘Chong Qing’ OR ‘He Bei’ OR ‘Shan Xi’ OR ‘Liao Ning’ OR ‘Ji Lin’ OR ‘Hei Long Jiang’ OR ‘Jiang Su’ OR ‘Zhe Jiang’ OR ‘An Hui’ OR ‘Fu Jian’ OR ‘Jiang Xi’ OR ‘Shan Dong’ OR ‘He Nan’ OR ‘Hu Bei’ OR ‘Hu Nan’ OR ‘Guang Dong’ OR ‘Hai Nan’ OR ‘Si Chuan’ OR ‘Gui Zhou’ OR ‘Yun Nan’ OR ‘Shaan Xi’ OR ‘Gan Su’ OR ‘Qing Hai’ OR ‘Nei Meng Gu’ OR ‘Guang Xi’ OR ‘Tibet’ OR ‘Xi Zang’ OR ‘Ning Xia’ OR ‘Xin Jiang’

OR

‘USA’ OR ‘US’ OR ‘United States’ OR ‘U.S.’ OR ‘U.S.A.’ OR ‘Alabama’ OR ‘AL’ OR ‘Ala.’ OR ‘Alaska’ OR ‘AK’ OR ‘Alaska’ OR ‘Arizona’ OR ‘AZ’ OR ‘Ariz.’ OR ‘Arkansas’ OR ‘AR’ OR ‘Ark.’ OR ‘California’ OR ‘CA’ OR ‘Calif.’ OR ‘Colorado’ OR ‘CO’ OR ‘Color.’ OR ‘Connecticut’ OR ‘CT’ OR ‘Conn.’ OR ‘Delaware’ OR ‘DE’ OR ‘Del.’ OR ‘Florida’ OR ‘FL’ OR ‘Fla.’ OR ‘Georgia’ OR ‘GA’ OR ‘Ga.’ OR ‘Hawaii’ OR ‘HI’ OR ‘Hawaii’ OR ‘Idaho’ OR ‘ID’ OR ‘Idaho’ OR ‘Illinois’ OR ‘IL’ OR ‘Ill.’ OR ‘Indiana’ OR ‘IN’ OR ‘Ind.’ OR ‘Iowa’ OR ‘IA’ OR ‘Iowa’ OR ‘Kansas’ OR ‘KS’ OR ‘Kan.’ OR ‘Kentucky’ OR ‘KY’ OR ‘Ky.’ OR ‘Louisiana’ OR ‘LA’ OR ‘La.’ OR ‘Maine’ OR ‘ME’ OR ‘Maine’ OR ‘Maryland’ OR ‘MD’ OR ‘Md.’ OR ‘Massachusetts’ OR ‘MA’ OR ‘Mass.’ OR ‘Michigan’ OR ‘MI’ OR ‘Mich.’ OR ‘Minnesota’ OR ‘MN’ OR ‘Minn.’ OR ‘Mississippi’ OR ‘MS’ OR ‘Miss.’ OR ‘Missouri’ OR ‘MO’ OR ‘Mo.’ OR ‘Montana’ OR ‘MT’ OR ‘Mont.’ OR ‘Nebraska’ OR ‘NE’ OR ‘Neb.’ OR ‘Nevada’ OR ‘NV’ OR ‘Nev.’ OR ‘New Hampshire’ OR ‘NH’ OR ‘N.H.’ OR ‘New Jersey’ OR ‘NJ’ OR ‘N.J.’ OR ‘New Mexico’ OR ‘NM’ OR ‘N.M.’ OR ‘New York’ OR ‘NY’ OR ‘N.Y.’ OR ‘North Carolina’ OR ‘NC’ OR ‘N.C.’ OR ‘North Dakota’ OR ‘ND’ OR ‘N.D.’ OR ‘Ohio’ OR ‘OH’ OR ‘Ohio’ OR ‘Oklahoma’ OR ‘OK’ OR ‘Okla.’ OR ‘Oregon’ OR ‘OR’ OR ‘Ore.’ OR ‘Pennsylvania’ OR ‘PA’ OR ‘Pa.’ OR ‘Rhode Island’ OR ‘RI’ OR ‘R.I.’ OR ‘South Carolina’ OR ‘SC’ OR ‘S.C.’ OR ‘South Dakota’ OR ‘SD’ OR ‘S.Dak.’ OR ‘Tennessee’ OR ‘TN’ OR ‘Tenn.’ OR ‘Texas’ OR ‘TX’ OR ‘Tex.’ OR ‘Utah’ OR ‘UT’ OR ‘Utah’ OR ‘Vermont’ OR ‘VT’ OR ‘V.T.’ OR ‘Virginia’ OR ‘VA’ OR ‘Va.’ OR ‘Washington’ OR ‘WA’ OR ‘Wash.’ OR ‘West Virginia’ OR ‘WV’ OR ‘W.Va.’ OR ‘Wisconsin’ OR ‘WI’ OR ‘Wis.’ OR ‘Wyoming’ OR ‘WY’ OR ‘Wyo.’):ff

**SinoMed**

("心肌梗"[标题] OR "心梗"[标题] OR "STEMI"[标题] OR "MI"[标题] OR "AMI"[标题] OR "NSTEMI"[标题] OR "ASTEMI"[标题])

AND

(PCI[标题] OR 介入[标题] OR 溶栓[标题] OR 尿激酶[标题] OR 尿活素[标题] OR 普佑克[标题] OR 链激酶[标题] OR 阿替普酶[标题] OR 瑞替普酶[标题] OR "t-pa" [标题] OR 纤溶酶原激活[标题] OR 爱通立[标题] OR 派通欣[标题] OR rPA[标题] OR 匹林[标题] OR 格雷[标题] OR 替格瑞洛[标题] OR 肝素[标题] OR 磺达肝癸[标题] OR 比伐卢定[标题] OR 急救[标题] OR 急诊[标题] OR 抗栓[标题] OR 抗凝[标题] OR 抗血小板[标题] OR 内科[标题] OR 速碧林[标题] OR 再灌注[标题] OR 临床治疗[标题] OR 药物治疗[标题])

**CNKI (China National Knowledge Infrastructure)**

(TI="心肌梗死" OR TI="心肌梗塞" OR TI="心梗" OR TI="STEMI" OR TI="MI" OR TI="AMI" OR TI="NSTEMI" OR TI="ASTEMI")

AND

(TI=PCI OR TI=介入 OR TI=溶栓 OR TI=尿激酶 OR TI=尿活素 OR TI=普佑克 OR TI=链激酶 OR TI=阿替普酶 OR TI=瑞替普酶 OR TI="t-pa" OR TI=纤溶酶原激活 OR TI=爱通立 OR TI=派通欣 OR TI=rPA OR TI=匹林 OR TI=格雷 OR TI=替格瑞洛 OR TI=肝素 OR TI=磺达肝癸 OR TI=比伐卢定 OR TI=急救 OR TI=急诊 OR TI=抗栓 OR TI=抗凝 OR TI=抗血小板 OR TI=内科 OR TI=速碧林 OR TI=再灌注 OR TI=临床治疗 OR TI=药物治疗)

**Wangfang**

( 题名: "心肌梗死" OR 题名: "心肌梗塞" OR 题名: "心梗" OR 题名: "STEMI" OR 题名: "MI" OR 题名: "AMI" OR 题名: "NSTEMI" OR 题名: "ASTEMI")

AND

( 题名: PCI OR 题名: 介入 OR 题名: 溶栓 OR 题名: 尿激酶 OR 题名: 尿活素 OR 题名: 普佑克 OR 题名: 链激酶 OR 题名: 阿替普酶 OR 题名: 瑞替普酶 OR 题名: "t-pa" OR 题名: 纤溶酶原激活 OR 题名: 爱通立 OR 题名: 派通欣 OR 题名: rPA OR 题名: 匹林 OR 题名: 格雷 OR 题名: 替格瑞洛 OR 题名: 肝素 OR 题名: 磺达肝癸 OR 题名: 比伐卢定 OR 题名: 急救 OR 题名: 急诊 OR 题名: 抗栓 OR 题名: 抗凝 OR 题名: 抗血小板 OR 题名: 内科 OR 题名: 速碧林 OR 题名: 再灌注 OR 题名: 临床治疗 OR 题名: 药物治疗)

**VIP**

T=(("心肌梗死" OR "心肌梗塞" OR "心梗" OR "STEMI" OR "MI" OR "AMI" OR "NSTEMI" OR "ASTEMI") AND (PCI OR 介入 OR 溶栓 OR 尿激酶 OR 尿活素 OR 普佑克 OR 链激酶 OR 阿替普酶 OR 瑞替普酶 OR "t-pa" OR 纤溶酶原激活 OR 爱通立 OR 派通欣 OR rPA OR 匹林 OR 格雷 OR 替格瑞洛 OR 肝素 OR 磺达肝癸 OR 比伐卢定 OR 急救 OR 急诊 OR 抗栓 OR 抗凝 OR 抗血小板 OR 内科 OR 速碧林 OR 再灌注 OR 临床治疗 OR 药物治疗))

(R="试验" OR R="安慰剂" OR R="对比" OR R="对照" OR R="比较" OR R="随机" OR R="硬币" OR R="抽签" OR R="骰子" OR R="双色球")

AND

("心肌梗死" OR "心肌梗塞" OR "心梗" OR "STEMI" OR "MI" OR "AMI" OR "NSTEMI" OR "ASTEMI")
