# Supplementary material for: Assessment of redundant randomized clinical trials among patients with ST segment elevation myocardial infarction
Source: BMC Med. 2023 Feb 24;21:69. doi: 10.1186/s12916-023-02749-2 (PMC9960404; doi:10.1186/s12916-023-02749-2)
Supplement: Supplementary file 2 — Additional file 2: Additional Method. The steps to calculate the confidence interval of extra MACEs are presented. [file 12916_2023_2749_MOESM2_ESM.docx]

Additional File 2 Additional Method

The following steps calculate the confidence interval of extra MACEs as an example.

**Step 1** For each RCT, calculate the risk difference of extra MACEs between the intervention group and the control group.

$$RD=\frac{{MACE}_{Control}}{N_{Control}}-\frac{{MACE}_{Intervention}}{N_{Intervention}}$$

*N_control_*: Number of patients analyzed in the control group.

*MACE_Control_*: Number of MACEs occurred among patients in the control group.

*N_Intervention_*: Number of patients analyzed in the intervention group.

*MACE_Intervention_*: Number of MACEs occurred among patients in the intervention group.

**Step 2** For each trial, calculate the standard error of risk difference of extra MACEs.

$$\sqrt{\frac{{RD}_{Intervention}(1-{RD}_{Intervention})}{{RD}_{Intervention}}-\frac{{RD}_{Control}(1-{RD}_{Control})}{{RD}_{Control}}}$$

**Step 3** Construct the distribution of each risk difference, assuming that the risk difference follows a normal distribution. The mean of the normal distribution is obtained from Step 1, while the standard deviation of the normal distribution is the standard error calculated from Step 2.

**Step 4** Repeat Step 1-3 for all the trials.

**Step 5** Draw a random value from the normal distribution of risk difference and calculate the extra MACEs based on that value.

**Step 6** Repeat Step 5 for all the trials and sum the extra MACEs together.

**Step 7** Repeat Step 5-6 for 1,000 times. Sort the 1,000 summed extra MACEs ascendingly. The 26^th^ value is the lower level of the confidence interval, while the 974^th^ value is the upper level. The 500^th^ value is the mean of summed extra MACEs.
