# Supplementary material for: Assessment of redundant randomized clinical trials among patients with ST segment elevation myocardial infarction
Source: BMC Med. 2023 Feb 24;21:69. doi: 10.1186/s12916-023-02749-2 (PMC9960404; doi:10.1186/s12916-023-02749-2)
Supplement: Supplementary file 3 — Additional file 3: Figure A1. The steps to select Eligible RCTs from bibliographic databases are presented. [file 12916_2023_2749_MOESM3_ESM.docx]

Additional File 3

Figure A1 Selection of Eligible RCTs

**Chinese Bibliographic Database**

**SinoMed N=24,122
CNKI N=42,246
Wanfang N=35,838
VIP N=22,669**

**English Bibliographic Databases**

**PubMed N=3,947
Embase N=4,111**

**Duplicate Records**

**N=81,257**

**Title and Abstract Screening**

**N=51,676**

**Ineligible Records**

**N=47,728**

**Full Text Screening**

**N=3,948**

**Ineligible Records N=2,960**

**Active Control N=386
Comorbidities N=18
Duplicate N=29
Ineligible Intervention N=209
No PDF N=25
Not RCT N=1685
Not STEMI N=495
Other Countries N=10
Protocol N=2
Unclear Intervention N=101**

**Eligible Records**

**N=988**

Abbreviations

CNKI: the China National Knowledge Infrastructure; RCT: Randomized Controlled Trials; STEMI: ST-segment Elevation Myocardial Infarction.
