# Supplementary material for: Assessment of redundant randomized clinical trials among patients with ST segment elevation myocardial infarction
Source: BMC Med. 2023 Feb 24;21:69. doi: 10.1186/s12916-023-02749-2 (PMC9960404; doi:10.1186/s12916-023-02749-2)
Supplement: Supplementary file 5 — Additional file 5: Figure A3. Cumulative Meta-Analysis for P2Y12 Receptor Inhibitors. This figure shows the result of a cumulative meta-analysis for RCTs assessing P2Y12 receptor inhibitors conducted in mainland China. Only the first 50 RCTs were analyzed due to the limit of Stata. [file 12916_2023_2749_MOESM5_ESM.docx]

Additional File 5

Figure A3 Cumulative Meta-Analysis for P2Y_12_ Receptor Inhibitors

This figure shows the result of a cumulative meta-analysis for RCTs assessing P2Y_12_ receptor inhibitors conducted in mainland China. Only the first 50 RCTs were analyzed due to the limit of Stata.
