# Supplementary material for: Assessment of redundant randomized clinical trials among patients with ST segment elevation myocardial infarction
Source: BMC Med. 2023 Feb 24;21:69. doi: 10.1186/s12916-023-02749-2 (PMC9960404; doi:10.1186/s12916-023-02749-2)
Supplement: Supplementary file 6 — Additional file 6: Figure A4. Cumulative Meta-Analysis for Statins. This figure shows the result of a cumulative meta-analysis for RCTs assessing statins conducted in mainland China. Only the first 50 RCTs were analyzed due to the limit of Stata. [file 12916_2023_2749_MOESM6_ESM.docx]

Additional File 6

Figure A4 Cumulative Meta-Analysis for Statins

This figure shows the result of a cumulative meta-analysis for RCTs assessing statins conducted in mainland China. Only the first 50 RCTs were analyzed due to the limit of Stata.
