# Supplementary material for: Assessment of redundant randomized clinical trials among patients with ST segment elevation myocardial infarction
Source: BMC Med. 2023 Feb 24;21:69. doi: 10.1186/s12916-023-02749-2 (PMC9960404; doi:10.1186/s12916-023-02749-2)
Supplement: Supplementary file 7 — Additional file 7: Figure A5. Cumulative Meta-Analysis for Anticoagulants. This figure shows the result of a cumulative meta-analysis for RCTs assessing anticoagulants conducted in mainland China. [file 12916_2023_2749_MOESM7_ESM.docx]

Additional File 7

Figure A5 Cumulative Meta-Analysis for Anticoagulants

This figure shows the result of a cumulative meta-analysis for RCTs assessing anticoagulants conducted in mainland China.
